# A New Entropy Function to Analyze Isentropic Processes of Ideal Gases with Variable Specific Heats

**DOI:** 10.3390/e24060746

**Published:** 2022-05-24

**Authors:** Yunus A. Çengel, Mehmet Kanoğlu

**Affiliations:** 1 Department of Mechanical Engineering, University of Nevada, Reno, NV 89557, USA; yunus.cengel@yahoo.com; 2 Department of Mechanical Engineering, Alanya Alaaddin Keykubat University, 07425 Antalya, Turkey

**Keywords:** thermodynamics, entropy, entropy functions, isentropic process, ideal gases, variable specific heats, second-law analysis

## Abstract

A new entropy function *s*^+^ is defined in terms of the existing entropy function *s*° and temperature as *s*^+^ = *s*° − *R* ln*T* to facilitate the analysis of isentropic processes of ideal gases with variable specific heats. The function s^+^ also makes it possible to calculate the entropy changes of ideal gases during processes when volume information is available instead of pressure information and the variation of specific heats with temperature is to be accounted for. The introduction of the function *s*^+^ eliminates the need to use the dimensionless isentropic functions relative pressure *P_r_* and relative specific volume *v_r_* of ideal gases and to tabulate their values. The *P_r_* and *v_r_* data are often confused with pressure and specific volume, with an adverse effect on the study of the second law of thermodynamics. The new *s*^+^ function nicely complements the existing *s*° function in entropy change calculations: the former is conveniently used when volume information is given while the latter is used when pressure information is available. Therefore, the introduction of the new entropy function *s*^+^ is expected to make a significant contribution to the thermodynamics education and research by streamlining entropy analysis of ideal gases.

## 1. Introduction

A good understanding of the fundamentals of thermodynamics is an essential part of an engineering education for most majors. Thermodynamics is usually considered a challenging subject by engineering students because of the abstractions involved. Entropy is an essential part of this course and students often struggle with making sense of it and using it in solving practical problems. It takes time to develop an intuitive understanding of the second-law concepts of entropy, entropy generation and isentropic efficiencies since, unlike energy, entropy is something which can be created out of nowhere. As a result, mastering entropy balance on processes is considered more difficult compared to the energy balance.

Entropy change calculations for real substances such as steam and the refrigerants are performed easily and conveniently by relying on the tabulated values of entropy. For ideal gases, we rely on the entropy change relations in the case of constant specific heats, and a combination of relations and tabulated *s*° data when the variation of specific heats with temperature is to be accounted for. In computerized calculations, entropy changes can be calculated easily by using property relations and built-in functions.

To facilitate the hand calculations for the analysis of isentropic processes of ideal gas with variable specific heats, the dimensionless functions relative pressure *P_r_* and relative specific volume *v_r_* are defined, and their values are listed in the ideal-gas tables. The values of *P_r_* and *v_r_* are determined by the numerical calculation of their defining integrals, and they have no physical meaning. Although the names and symbols imply otherwise, *P_r_* and *v_r_* have to relation to pressure or specific volume. The use of these terms adds confusion and impacts thermodynamic education negatively.

Costa [1] identified and discussed the reasons why thermodynamics is viewed as a difficult subject by engineering students. A survey of college students indicated significant challenges concerning students’ understanding of thermodynamic processes, the first and second laws, and entropy [2]. Mulop et al. [3] acknowledged the difficulties of students learning thermodynamics globally and presented a review and analysis of the different approaches to supporting students’ learning of thermodynamics. Goyings and Arnas [4] noted some difficulties in teaching the second law and the related concepts of entropy and exergy, and proposed an alternative way of teaching thermodynamics. 

Yang et al. [5] proposed some methods to address some of the challenges associated with the heat transfer and thermodynamics education. Other researchers proposed alternative methods to deal with difficulties involved in learning of thermodynamics and entropy. An alternative to the Carnot–Clausius approach for introducing entropy and the second law of thermodynamics is outlined, and entropy is introduced as a nonequilibrium property [6]. Acevedo et al. [7] presented a new educational package based on e-learning concerning engineering thermodynamics processes, combustion, energy, and entropy analysis. Smith [8] illustrated how thermodynamic energy, entropy, and the second law of thermodynamics can be intuitively related to student’s experiences. Recently Çengel [9] addressed the confusion caused by the use of entropy and information interchangeably, and attempted to put things into proper perspective.

There are a number of studies in the literature on the significance of using variable specific heats for the analysis of energy conversion systems involving ideal gases. Many studies indicate that the effects of using variable specific heats for the working fluid in the air-standard cycles such as the diesel cycle, the dual cycle, and the Brayton cycle performance are significant and they should be considered in cycle analysis [10,11,12,13,14]. 

In this paper, we introduce a new entropy function *s*^+^ to be used in entropy analysis of ideal gases when the variation of specific heats with temperature are considered. The new function is used in both the entropy change calculations and isentropic process analysis of ideal gases. The introduction of this new entropy term *s*^+^ complements the existing entropy function *s*° and eliminates the need to define and use of relative pressure *P_r_* and relative specific volume *v_r_* in the analysis of isentropic processes of ideal gases. 

First, we present the existing coverage for the entropy change and isentropic processes of ideal gases as found in most thermodynamics textbooks [15,16,17]. Then, we define the new entropy term *s*^+^ and provide a property table for air which includes the *s*^+^ values. We demonstrate the use of *s*^+^ by solving some sample problems using both the existing approach and the new approach. Finally, we discuss the advantages of the new *s*^+^ term with respect to the existing coverage of entropy. 

## 2. Entropy Change of Ideal Gases

The coverage presented here is based on Çengel et al. [15], but is also available in most thermodynamics textbooks. The differential change of entropy is expressed in terms of other properties as

(1) $$ds=\frac{du}{T}+\frac{Pdv}{T}$$

(2) $$ds=\frac{dh}{T}-\frac{vdP}{T}$$

where *s* is entropy, *u* is internal energy, *h* is enthalpy, *T* is temperature, *P* is pressure, and *v* is the specific volume. Substituting the property relations *du* = *c_v_ dT*, *dh* = *c_p_ dT*, and *P* = *RT*/*v* where *R* is the gas constant into the equations above and integrating them for a process between states 1 and 2 gives

(3) $$s_{2}-s_{1}=\int_{1}^{2}c_{p}(T)\frac{dT}{T}-R\ln\frac{P_{2}}{P_{1}}$$

(4) $$s_{2}-s_{1}=\int_{1}^{2}c_{v}(T)\frac{dT}{T}+R\ln\frac{v_{2}}{v_{1}}$$

The specific heats of ideal gases, with the exception of monatomic gases, depend on temperature, and performing the integrals in the equations above requires the use of *c_v_*(*T*) and *c_p_*(*T*) functions, which is not practical for hand calculations. Assuming constant specific heats at some average value for simplicity gives the following approximate relations:

(5) $$s_{2}-s_{1}=c_{v,\mathrm{avg}}\ln\frac{T_{2}}{T_{1}}+R\ln\frac{v_{2}}{v_{1}}$$

(6) $$s_{2}-s_{1}=c_{p,\mathrm{avg}}\ln\frac{T_{2}}{T_{1}}-R\ln\frac{P_{2}}{P_{1}}$$

For better accuracy, the variation of specific heats with temperature needs be accounted for. In computerized calculations, this can be easily done by using the built-in functions for properties and numerical integration schemes. For hand calculations, it is convenient to perform these integrals once and tabulate the results. For this purpose, it is common to choose absolute zero as the reference temperature and define a function *s*° as

(7) $$s^{{}^{\circ}}=\int_{0}^{T}c_{p}(T)\frac{dT}{T}$$

Then the integral in Equation (3) is simply $s_{2}^{{}^{\circ}}-s_{1}^{{}^{\circ}}$, and Equation (3) becomes

(8) $$s_{2}-s_{1}=s_{2}^{{}^{\circ}}-s_{1}^{{}^{\circ}}-R\ln\frac{P_{2}}{P_{1}}$$

Now, noting that $c_{v}=c_{p}-R$ for ideal gases and utilizing Equation (7), the integral in Equation (4) can be expressed as

$$\begin{array}{cc} \int_{1}^{2}c_{v}(T)\frac{dT}{T} & =\int_{1}^{2}\left[c_{p}(T)-R\right]\frac{dT}{T}\\ & =s_{2}^{{}^{\circ}}-s_{1}^{{}^{\circ}}-R\ln\frac{T_{2}}{T_{1}}\\ & =(s_{2}^{{}^{\circ}}-R\ln T_{2})-(s_{1}^{{}^{\circ}}-R\ln T_{1})\\ & =s_{2}^{+}-s_{1}^{+} \end{array}$$

where the function *s*^+^ is defined as

(9) $$s^{+}=s^{{}^{\circ}}-R\ln T$$

That is, the value of *s*^+^ of an ideal at a given temperature can be determined by subtracting *R* ln*T* from the value of *s*° at the same temperature. Then, the entropy change of an ideal gas can also be expressed as

(10) $$s_{2}-s_{1}=s_{2}^{+}-s_{1}^{+}+R\ln\frac{v_{2}}{v_{1}}$$

This is equivalent to

$$s_{2}-s_{1}=s_{2}^{{}^{\circ}}-s_{1}^{{}^{\circ}}-R\ln\frac{T_{2}}{T_{1}}+R\ln\frac{v_{2}}{v_{1}}$$

The definition of the function *s*^+^ and the new entropy change relation in Equation (10) are important additions to the literature since they enable the calculation of entropy change of ideal gases when the volume information is given instead of pressure information. This way, tedious iterations are avoided in hand calculations. 

It is clear that *s*° and *s*^+^ are functions of temperature alone, and their values are zero at absolute zero temperature. The values of *s*^+^ are calculated from its definition $s^{+}=s^{{}^{\circ}}-R\ln T$ for air at various temperatures and the results are listed in Table 1 together with other properties of air.

It is noted that, unlike internal energy and enthalpy, the entropy of an ideal gas varies with specific volume or pressure as well as the temperature. Therefore, entropy cannot be tabulated as a function of temperature alone. The *s*° and *s*^+^ values in the tables account for the temperature dependence of entropy.

## 3. Isentropic Processes of Ideal Gases

Several relations for the isentropic processes of ideal gases can be obtained by setting the entropy-change relations equal to zero. When this is done for the case of constant specific heats, we obtain

*Tv ^k^*^− 1^ = constant

*TP*^(1 − *k*)/*k*^ = constant

*Pv ^k^* = constant

Isentropic relations of ideal gases for the case of variable specific heats are obtained by setting Equations (8) and (10) equal to zero, yielding

(11) $$s_{2}^{{}^{\circ}}=s_{1}^{{}^{\circ}}+R\ln\frac{P_{2}}{P_{1}}$$

(12) $$s_{2}^{+}=s_{1}^{+}-R\ln\frac{v_{2}}{v_{1}}$$

These two equations are equivalent, and one can be derived from the other. They vary only in convenience: Equation (11) should be used when pressure information is given for a process and Equation (12) should be used when volume information is given to avoid iteration.

Next, we provide definitions for relative pressure *P_r_* and relative specific volume *v_r_* for the completeness of the treatment. First, we rearrange Equation (11) as

$$\frac{P_{2}}{P_{1}}=\exp\frac{s_{2}^{{}^{\circ}}-s_{1}^{{}^{\circ}}}{R}$$

or

$$\frac{P_{2}}{P_{1}}=\frac{\exp(s_{2}^{{}^{\circ}}/R)}{\exp(s_{1}^{{}^{\circ}}/R)}$$

The quantity *C*_1_ exp(*s*°/*R*) is defined as the relative pressure *P_r_* where *C*_1_ is a constant with a proper unit. With this definition, the last relation becomes

(13) $$\frac{P_{2}}{P_{1}}=\frac{P_{r2}}{P_{r1}}$$

Note that the relative pressure *P_r_* is a *dimensionless* quantity that is a function of temperature only since *s*° depends on temperature alone. Therefore, values of *P_r_* can be tabulated against temperature. This is done for air in Table 1. Sometimes, specific volume ratios are given instead of pressure ratios. This is particularly the case when automotive engines are analyzed. In such cases, one needs to work with volume ratios. Therefore, we define another quantity related to specific volume ratios for isentropic processes. This is done by utilizing the ideal-gas relation and Equation (13):

$$\frac{P_{1}v_{1}}{T_{1}}=\frac{P_{2}v_{2}}{T_{2}}\to \frac{v_{2}}{v_{1}}=\frac{T_{2}P_{1}}{T_{1}P_{2}}=\frac{T_{2}P_{r1}}{T_{1}P_{r2}}=\frac{T_{2}/P_{r2}}{T_{1}/P_{r1}}$$

The quantity *C*_2_*T*/*P_r_* or *C*_2_*T*/exp(*s*°/*R*) is a function of temperature only and is defined as relative specific volume *v_r_* where *C*_2_ is a constant with a proper unit. Thus,

(14) $$\frac{v_{2}}{v_{1}}=\frac{v_{r2}}{v_{r1}}$$

Equations (13) and (14) are used for the isentropic processes of ideal gases with variable specific heats. The introduction of the function *s*^+^ renders the definitions of relative pressure *P_r_* and relative specific volume *v_r_* obsolete. 

We should note that the relative pressure and relative specific volume are useful when it comes to problems where temperatures are known but pressure or volume needs to be determined. For example, assume we need to calculate final pressure (*P*_2_) during an isentropic process when initial temperature and pressure (*T*_1_ and *P*_1_) and final temperature (*T*_2_) are given. The final pressure can be determined easily using the relation (*P*_2_/*P*_1_ = *P_r_*_2_/*P_r_*_1_) after reading *P_r_*_1_ and *P_r_*_2_ values from Table 1 at the given temperatures *T*_1_ and *T*_2_. The final pressure can also be determined using the relation [$s_{2}^{{}^{\circ}}=s_{1}^{{}^{\circ}}+R\ln(P_{2}/P_{1})$] after reading $s_{1}^{{}^{\circ}}$ and $s_{2}^{{}^{\circ}}$ from Table 1 at *T*_1_ and *T*_2_. This method also involves a single relation but requires more operations due to the gas constant *R* and the exponential term. This particular case has little practical importance since the exit pressure is normally taken to be the same for the actual and isentropic processes involving work-consuming and work-producing devices such as turbines and compressors. 

Next, we solve two example problems using the existing conventional approach as found in most thermodynamics books and the new approach proposed in this paper based on the new entropy term *s*^+^. The first example deals with the calculation of the entropy change of air and the second example deals with the analysis of the ideal Otto cycle that includes two isentropic processes of the air. 

## 4. Example Problem 1 with the Existing Approach

A 0.75 kg amount of air at 290 K is compressed in a piston-cylinder device from a volume of 0.5 m^3^ to 0.2 m^3^ and a final temperature of 400 K. Entropy change of air is determined considering variation of specific heats with temperature. 

The only relation we have for the entropy change of an ideal gas under variable specific heat assumption is Equation (8):

$$s_{2}-s_{1}=s_{2}^{{}^{\circ}}-s_{1}^{{}^{\circ}}-R\ln\frac{P_{2}}{P_{1}}$$

But we do not have pressure information at the initial and final states. Therefore, we need to first determine pressure values using the ideal-gas relation:

$$\begin{array}{l} P_{1}V_{1}=mRT_{1}\overset{}{\to }P_{1}(0.5{\;m}^{3})=(0.75\;\mathrm{kg})(0.287\;\mathrm{kJ}/\mathrm{kg}\cdot K)(290\;K)\overset{}{\to }P_{1}=124.8\;\mathrm{kPa}\\ P_{2}V_{2}=mRT_{2}\overset{}{\to }P_{2}(0.2{\;m}^{3})=(0.75\;\mathrm{kg})(0.287\;\mathrm{kJ}/\mathrm{kg}\cdot K)(650\;K)\overset{}{\to }P_{2}=699.6\;\mathrm{kPa} \end{array}$$

We obtain *s*° values from Table 1:

$$T_{1}=290\;K\;\;\overset{}{\to }\;\;s_{1}^{{}^{\circ}}=1.66802\;\mathrm{kJ}/\mathrm{kg}\cdot K$$

$$T_{2}=650\;K\;\;\overset{}{\to }\;\;s_{2}^{{}^{\circ}}=2.49364\;\mathrm{kJ}/\mathrm{kg}\cdot K$$

Substituting,

$$\begin{array}{cc} s_{2}-s_{1} & =s_{2}^{{}^{\circ}}-s_{1}^{{}^{\circ}}-R\ln\frac{P_{2}}{P_{1}}\\ & =(2.49364-1.66802)\;\mathrm{kJ}/\mathrm{kg}\cdot K-(0.287\;\mathrm{kJ}/\mathrm{kg}\cdot K)\ln\frac{699.6\;\mathrm{kPa}}{124.8\;\mathrm{kPa}}\\ & =0.331\;\mathrm{kJ}/\mathrm{kg}\cdot K \end{array}$$

The entropy change is

$$\Delta S=m(s_{2}-s_{1})=(0.75\;\mathrm{kg})(0.331\;\mathrm{kJ}/\mathrm{kg}\cdot K)=0.248\;\mathrm{kJ}/K$$

## 5. Example Problem 1 with the New Approach

Now, we solve the same problem with the new approach by utilizing the *s*^+^ term. Since volume information is given, the entropy change can be determined directly from Equation (10):

$$s_{2}-s_{1}=s_{2}^{+}-s_{1}^{+}+R\ln\frac{V_{2}}{V_{1}}$$

We obtain *s*^+^ values from Table 1:

$$T_{1}=290\;K\;\;\overset{}{\to }\;\;s_{1}^{+}=0.04076\;\mathrm{kJ}/\mathrm{kg}\cdot K$$

$$T_{2}=650\;K\;\;\overset{}{\to }\;\;s_{2}^{+}=0.63475\;\mathrm{kJ}/\mathrm{kg}\cdot K$$

Substituting,

$$\begin{array}{cc} s_{2}-s_{1} & =s_{2}^{+}-s_{1}^{+}+R\ln\frac{V_{2}}{V_{1}}\\ & =(0.63475-0.04076)\;\mathrm{kJ}/\mathrm{kg}\cdot K+(0.287\;\mathrm{kJ}/\mathrm{kg}\cdot K)\ln\frac{0.2{\;m}^{3}}{0.5{\;m}^{3}}\\ & =0.331\;\mathrm{kJ}/\mathrm{kg}\cdot K \end{array}$$

The entropy change is

$$\Delta S=m(s_{2}-s_{1})=(0.75\;\mathrm{kg})(0.331\;\mathrm{kJ}/\mathrm{kg}\cdot K)=0.248\;\mathrm{kJ}/K$$

## 6. Example Problem 2 with the Existing Approach

An ideal Otto cycle has a compression ratio of 8. At the beginning of the compression process, air is at 95 kPa and 27 °C, and 750 kJ/kg of heat is transferred to air during the constant-volume heat-addition process. Taking into account the variation of specific heats with temperature, the pressure and temperature at the end of the heat-addition process, the network output, and the thermal efficiency are determined. 

**Figure 1:**
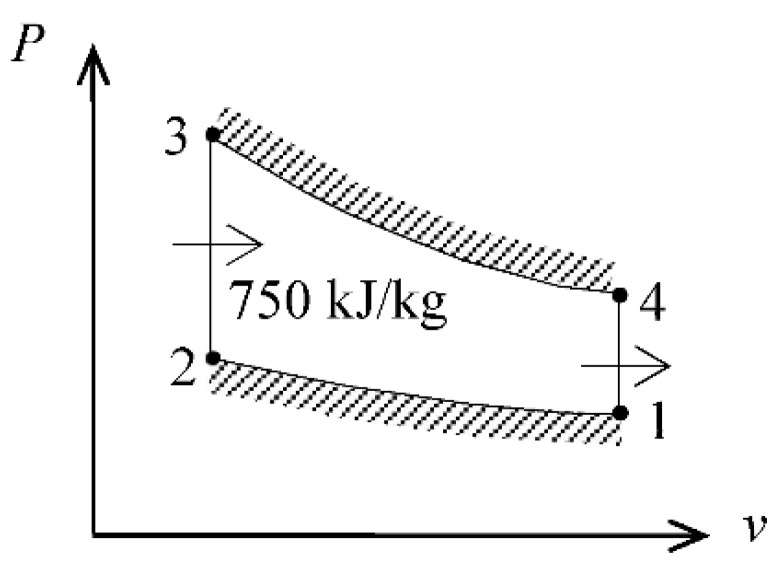
*P*-*v* diagram of the Otto cycle.

We solve this problem using the properties in Table 1. We use air-standard assumptions and neglect kinetic and potential energy changes. The gas constant of air is *R* = 0.287 kJ/kg·K. The properties of air are given in Table 1. A *P*-*v* diagram of the cycle is given in Figure 1.

Process 1-2: isentropic compression
  $$T_{1}=300\;K\;\;\overset{}{\to }\;\;\begin{array}{c} \begin{array}{l} u_{1}=214.07\;\mathrm{kJ}/\mathrm{kg}\\ v_{r1}=621.2 \end{array} \end{array}$$
  $$v_{r2}=\frac{v_{2}}{v_{1}}v_{r1}=\frac{1}{r}v_{r1}=\frac{1}{8}\left(621.2\right)=77.65\;\;\to \;\;\;\begin{array}{c} \begin{array}{l} T_{2}=673.1\;K\\ u_{2}=491.2\;\mathrm{kJ}/\mathrm{kg} \end{array} \end{array}$$
  $$\frac{P_{2}v_{2}}{T_{2}}=\frac{P_{1}v_{1}}{T_{1}}\;\;\overset{}{\to }\;\;P_{2}=\frac{v_{1}}{v_{2}}\frac{T_{2}}{T_{1}}P_{1}=\left(8\right)\left(\frac{673.1\;K}{300\;K}\right)\left(95\;\mathrm{kPa}\right)=1705\;\mathrm{kPa}$$
Process 2-3: *v* = constant heat addition
  $$q_{23,\mathrm{in}}=u_{3}-u_{2}\overset{}{\to }u_{3}=u_{2}+q_{23,\mathrm{in}}=491.2+750=1241.2\;\mathrm{kJ}/\mathrm{kg}\;\;\overset{}{\to }\;\;\begin{array}{c} \begin{array}{l} T_{3}=1539\;K\\ v_{r3}=6.588 \end{array} \end{array}$$
  $$\frac{P_{3}v_{3}}{T_{3}}=\frac{P_{2}v_{2}}{T_{2}}\;\;\overset{}{\to }\;\;P_{3}=\frac{T_{3}}{T_{2}}P_{2}=\left(\frac{1539\;K}{673.1\;K}\right)\left(1705\;\mathrm{kPa}\right)=3898\;\mathrm{kPa}$$
Process 3-4: isentropic expansion
  $$v_{r4}=\frac{v_{1}}{v_{2}}v_{r3}=rv_{r3}=\left(8\right)\left(6.588\right)=52.70\;\;\overset{}{\to }\;\;\begin{array}{c} \begin{array}{l} T_{4}=774.5\;K\\ u_{4}=571.69\;\mathrm{kJ}/\mathrm{kg} \end{array} \end{array}$$
Process 4-1: *v* = constant heat rejection
  $$q_{\mathrm{out}}=u_{4}-u_{1}=571.69-214.07=357.62\;\mathrm{kJ}/\mathrm{kg}$$
The network output and the thermal efficiency are
  $$w_{\mathrm{net},\mathrm{out}}=q_{\mathrm{in}}-q_{\mathrm{out}}=750-357.62=392.4\;\mathrm{kJ}/\mathrm{kg}$$
  $$\eta_{\mathrm{th}}=\frac{w_{\mathrm{net},\mathrm{out}}}{q_{\mathrm{in}}}=\frac{392.4\;\mathrm{kJ}/\mathrm{kg}}{750\;\mathrm{kJ}/\mathrm{kg}}=0.523=52.3\%$$

## 7. Example Problem 2 with the New Approach

We solve the same Otto cycle problem using the new approach by using the newly defined *s*^+^ term. In the solution we use the values in Table 1.

Process 1-2: isentropic compression
  $$T_{1}=300\;K\;\;\overset{}{\to }\;\;\begin{array}{c} \begin{array}{l} u_{1}=214.07\;\mathrm{kJ}/\mathrm{kg}\\ s_{1}^{+}=0.06504\;\mathrm{kJ}/\mathrm{kg}\cdot K \end{array} \end{array}$$
  $$s_{2}^{+}=s_{1}^{+}-R\ln\frac{v_{2}}{v_{1}}=0.06504-(0.287)\ln(1/8)=0.66184\;\mathrm{kJ}/\mathrm{kg}\cdot K\;\overset{}{\to }\;\;\begin{array}{c} \begin{array}{l} T_{2}=673.1\;K\\ u_{2}=491.2\;\mathrm{kJ}/\mathrm{kg} \end{array} \end{array}$$
  $$\frac{P_{2}v_{2}}{T_{2}}=\frac{P_{1}v_{1}}{T_{1}}\;\;\overset{}{\to }\;\;P_{2}=\frac{v_{1}}{v_{2}}\frac{T_{2}}{T_{1}}P_{1}=\left(8\right)\left(\frac{673.1\;K}{300\;K}\right)\left(95\;\mathrm{kPa}\right)=1705\;\mathrm{kPa}$$
Process 2-3: *v* = constant heat addition
  $$q_{23,\mathrm{in}}=u_{3}-u_{2}\overset{}{\to }u_{3}=u_{2}+q_{23,\mathrm{in}}=491.2+750=1241.2\;\mathrm{kJ}/\mathrm{kg}\;\overset{}{\to }\;\;\begin{array}{c} \begin{array}{l} T_{3}=1539\;K\\ s_{3}^{+}=1.37006\;\mathrm{kJ}/\mathrm{kg}\cdot K \end{array} \end{array}\;\frac{P_{3}v_{3}}{T_{3}}=\frac{P_{2}v_{2}}{T_{2}}\;\;\overset{}{\to }\;\;P_{3}=\frac{T_{3}}{T_{2}}P_{2}=\left(\frac{1539\;K}{673.1\;K}\right)\left(1705\;\mathrm{kPa}\right)=3898\;\mathrm{kPa}$$
Process 3-4: isentropic expansion
  $$s_{4}^{+}=s_{3}^{+}-R\ln\frac{v_{4}}{v_{3}}=1.37006-(0.287)\ln(8)=0.77326\;\mathrm{kJ}/\mathrm{kg}\cdot K\overset{}{\to }\;\;\begin{array}{c} \begin{array}{l} T_{4}=774.5\;K\\ u_{4}=571.69\;\mathrm{kJ}/\mathrm{kg} \end{array} \end{array}$$
Process 4-1: *v* = constant heat rejection
  $$q_{\mathrm{out}}=u_{4}-u_{1}=571.69-214.07=357.62\;\mathrm{kJ}/\mathrm{kg}$$
  $$w_{\mathrm{net},\mathrm{out}}=q_{\mathrm{in}}-q_{\mathrm{out}}=750-357.62=392.4\;\mathrm{kJ}/\mathrm{kg}$$
The network output and the thermal efficiency are
  $$\eta_{\mathrm{th}}=\frac{w_{\mathrm{net},\mathrm{out}}}{q_{\mathrm{in}}}=\frac{392.4\;\mathrm{kJ}/\mathrm{kg}}{750\;\mathrm{kJ}/\mathrm{kg}}=0.523=52.3\%$$

## 8. Discussion

The solution of the two example problems using the existing approach and the new approach clearly show the convenience of the new approach in entropy analysis of ideal gases with variable specific heat treatment. The advantages of defining the new entropy term *s*^+^ can be explained as follows.

The concept of relative pressure *P_r_* and relative specific volume *v_r_* are eliminated. Entropy is already a challenging subject in thermodynamics and forcing students to learn and use these *P_r_* and *v_r_* functions makes it even more difficult. These functions were introduced a long time ago to allow isentropic process calculations when people did not have electronic calculators and had to use logarithmic tables or slide rulers.

Table 1 is an abbreviated version of air table that exists in most thermodynamics textbooks. It includes one column for the entropy term *s*° and two more columns for relative pressure *P_r_* and relative specific volume *v_r_*. With the introduction of the *s*^+^ term, a new column for the *s*^+^ term is added but two columns for *P_r_* and *v_r_* are eliminated. With this change, students can perform the same entropy change and isentropic process calculations for air under variable specific heat treatment by dealing with a smaller amount of data in the air table. 

In the existing system, for the entropy change of ideal gases under variable specific heat treatment, only one equation (Equation (8)) is available. If temperature and pressure information is given in the initial and final states (or inlet and exit states), the student can easily calculate entropy change using Equation (8). However, if temperature and volume information is given in the initial and final states, there is no relation available. In this case, the student needs to first find pressure information at the initial and final states and then use Equation (8) to calculate the entropy change (see Example Problem 1 using the existing approach). With the introduction of the *s*^+^ term and its inclusion in the air table, the student can directly use Equation (10) to determine entropy change, as shown in the solution of Example Problem 1 using the new approach.

In the existing system, for the isentropic processes of ideal gases under variable specific heat treatment, three equations (Equations (11), (13) and (14)) are available. Equations (11) and (13) are equivalent as they are both used when pressure information is given. In the new approach, both the relative pressure *P_r_* and the relative specific volume *v_r_* are eliminated and their values in the air table are removed. With the definition of the *s*^+^ term and the addition of its values in the air table, a student only needs two equations (Equations (11) and (12)) to solve isentropic process problems. Equation (11) is used when pressure information is given and Equation (12) is used when volume information is given.

The entropy terms *s*° and *s*^+^ have some physical sense but not *P_r_* and *v_r_* functions. The change in *s*° represents the temperature-dependent part of the entropy change when the pressure information is available (Equation (8)). Similarly, the change in *s*^+^ represents the temperature-dependent part of the entropy change when the volume information is available (Equation (10)). The *P_r_* and *v_r_* functions are dimensionless quantities but the *s*° and *s*^+^ terms have the same unit as entropy.

An additional advantage of eliminating the relative pressure *P_r_* and relative specific volume *v_r_* is that these terms will no longer be confused with pressure and specific volume as well as the reduced pressure *P_R_* and pseudo-reduced specific volume *v_R_* terms used in the compressibility factor calculations.

## 9. Conclusions

The definition of a new entropy term *s*^+^ and its inclusion in ideal-gas tables along with the existing term *s*° allows a convenient way of calculating entropy change of ideal gases when variation of specific heats with temperature is accounted for. It also allows the calculation of final state properties when isentropic processes of ideal gases are considered. As a result, the need for the definitions of relative pressure *P_r_* and relative specific volume *v_r_* is eliminated and the values of *P_r_* and *v_r_* are removed from ideal-gas tables. Entropy is viewed as a challenging subject by most students of thermodynamics. Thus, the introduction of the new entropy term *s*^+^ is expected to provide considerable convenience and ease in the study of entropy and the second law analysis of energy systems.

## Figures and Tables

**Table 1:** Ideal-gas properties of air. Source: Çengel et al. [15] supplemented with *s*^+^ values. (With the introduction of *s*^+^, the *P_r_* and *v_r_* columns are no longer needed).

*T*, K	*h*, kJ/kg	*u*, kJ/kg	*P_r_*	*v_r_*	*s*°, kJ/kg·K	*s*^+^, kJ/kg·K
200	199.97	142.56	0.3363	1707.0	1.29559	−0.22503
220	219.97	156.82	0.4690	1346.0	1.39105	−0.15692
240	240.02	171.13	0.6355	1084.0	1.47824	−0.09470
260	260.09	185.45	0.8405	887.8	1.55848	−0.03744
280	280.13	199.75	1.0889	738.0	1.63279	0.01561
290	290.16	206.91	1.2311	676.1	1.66802	0.04076
300	300.19	214.07	1.3860	621.2	1.70203	0.06504
320	320.29	228.42	1.7375	528.6	1.76690	0.11139
340	340.42	242.82	2.149	454.1	1.82790	0.15499
360	360.58	257.24	2.626	393.4	1.88543	0.19612
380	380.77	271.69	3.176	343.4	1.94001	0.23518
400	400.98	286.16	3.806	301.6	1.99194	0.27239
420	421.26	300.69	4.522	266.6	2.04142	0.30787
440	441.61	315.30	5.332	236.8	2.08870	0.34180
460	462.02	329.97	6.245	211.4	2.13407	0.37441
480	482.49	344.70	7.268	189.5	2.17760	0.40572
500	503.02	359.49	8.411	170.6	2.21952	0.43593
540	544.35	389.34	11.10	139.7	2.29906	0.49338
580	586.04	419.55	14.38	115.7	2.37348	0.54729
600	607.02	434.78	16.28	105.8	2.40902	0.57310
640	649.22	465.50	20.64	88.99	2.47716	0.62272
680	691.82	496.62	25.85	75.50	2.54175	0.66991
700	713.27	512.33	28.80	69.76	2.57277	0.69261
740	756.44	544.02	35.50	59.82	2.63280	0.73669
780	800.03	576.12	43.35	51.64	2.69013	0.77891
800	821.95	592.30	47.75	48.08	2.71787	0.79939
840	866.08	624.95	57.60	41.85	2.77170	0.83921
880	910.56	657.95	68.98	36.61	2.82344	0.87760
900	932.93	674.58	75.29	34.31	2.84856	0.89627
940	977.92	708.08	89.28	30.22	2.89748	0.93271
980	1023.25	741.98	105.2	26.73	2.94468	0.96795
1000	1046.04	758.94	114.0	25.17	2.96770	0.98517
1040	1091.85	793.36	133.3	23.29	3.01260	1.01882
1100	1161.07	845.33	167.1	18.896	3.07732	1.06744
1140	1207.57	880.35	193.1	16.946	3.11883	1.09870
1200	1277.79	933.33	238.0	14.470	3.17888	1.14403
1240	1324.93	968.95	272.3	13.069	3.21751	1.17325
1300	1395.97	1022.82	330.9	11.275	3.27345	1.21563
1340	1443.6	1058.94	375.3	10.247	3.30959	1.24307
1400	1515.42	1113.52	450.5	8.919	3.36200	1.28291
1440	1563.51	1150.13	506.9	8.153	3.39586	1.30868
1500	1635.97	1205.41	601.9	7.152	3.44516	1.34627
1540	1684.51	1242.43	672.8	6.569	3.47712	1.37067
1600	1757.57	1298.30	791.2	5.804	3.52364	1.40622
1640	1806.46	1335.72	878.9	5.355	3.55381	1.42931
1700	1880.1	1392.7	1025	4.761	3.5979	1.46308
1750	1941.6	1439.8	1161	4.328	3.6336	1.49046
1800	2003.3	1487.2	1310	3.994	3.6684	1.51718
1850	2065.3	1534.9	1475	3.601	3.7023	1.54322
1900	2127.4	1582.6	1655	3.295	3.7354	1.56866
1950	2189.7	1630.6	1852	3.022	3.7677	1.59351
2000	2252.1	1678.7	2068	2.776	3.7994	1.61794
2050	2314.6	1726.8	2303	2.555	3.8303	1.64175
2100	2377.7	1775.3	2559	2.356	3.8605	1.66504
2150	2440.3	1823.8	2837	2.175	3.8901	1.68788
2200	2503.2	1872.4	3138	2.012	3.9191	1.71029
2250	2566.4	1921.3	3464	1.864	3.9474	1.73214
